# Significance of Complement System in Ischemic Stroke: A Comprehensive Review

**DOI:** 10.14336/AD.2019.0119

**Published:** 2019-04-01

**Authors:** Yuanyuan Ma, Yanqun Liu, Zhijun Zhang, Guo-Yuan Yang

**Affiliations:** ^1^Department of Neurology, Ruijin Hospital, School of Medicine, Shanghai Jiao Tong University, Shanghai, China; ^2^Med-X Research Institute and School of Biomedical Engineering, Shanghai Jiao Tong University, Shanghai, China; ^3^Department of Neurology, Changhai Hospital, Second Military Medical University, Shanghai, China

**Keywords:** brain, C3a, C5a, complement, ischemic stroke

## Abstract

The complement system is an essential part of innate immunity, typically conferring protection via eliminating pathogens and accumulating debris. However, the defensive function of the complement system can exacerbate immune, inflammatory, and degenerative responses in various pathological conditions. Cumulative evidence indicates that the complement system plays a critical role in the pathogenesis of ischemic brain injury, as the depletion of certain complement components or the inhibition of complement activation could reduce ischemic brain injury. Although multiple candidates modulating or inhibiting complement activation show massive potential for the treatment of ischemic stroke, the clinical availability of complement inhibitors remains limited. The complement system is also involved in neural plasticity and neurogenesis during cerebral ischemia. Thus, unexpected side effects could be induced if the systemic complement system is inhibited. In this review, we highlighted the recent concepts and discoveries of the roles of different kinds of complement components, such as C3a, C5a, and their receptors, in both normal brain physiology and the pathophysiology of brain ischemia. In addition, we comprehensively reviewed the current development of complement-targeted therapy for ischemic stroke and discussed the challenges of bringing these therapies into the clinic. The design of future experiments was also discussed to better characterize the role of complement in both tissue injury and recovery after cerebral ischemia. More studies are needed to elucidate the molecular and cellular mechanisms of how complement components exert their functions in different stages of ischemic stroke to optimize the intervention of targeting the complement system.

## 1. Introduction

Stroke is the most common cerebral vascular disease and a leading cause of permanent disability and death worldwide today [1, 2]. The traditional definition of stroke is based on the sudden onset of the loss of focal neurological function due to infarction or hemorrhage in the relevant portion of the brain [3]. There are two main types of stroke: ischemic stroke and hemorrhagic stroke. The majority of strokes are ischemic (70-80%) and have various mechanisms and management approaches compared to hemorrhagic strokes [4]. Over the past several decades, there has been substantial progress in the understanding of the pathophysiology of ischemic stroke. Following an ischemic stroke attack, patients could show blood flow restoration in the infarct region, either endogenous thrombolytic system activation or exogenous thrombolytic therapy. Cerebral blood flow reperfusion initiates a cascade of pathophysiological events that could aggravate brain tissue damage, leading to more severe neurological function and cognitive deficits [5]. Currently, the molecular mechanism of cerebral ischemia/reperfusion injury is not completely elucidated; however, complement activation and its products have been found to be strongly implicated in one of the putative mechanisms [6]. A large set of data showed that complement inhibition could improve the outcomes of ischemic stroke in many animal models [7]. The involvement of the complement system in the pathogenesis of human ischemic stroke has been demonstrated by findings of local deposits of various complement components in the post-stroke brain tissue [8, 9]. Numerous approaches targeting complement were tried to reduce ischemic damage, including inhibition of complement component 1 (C1), complement component 3 (C3), complement component 5 (C5), and membrane attack complex (MAC, C5b-9). Cobra venom factor (CVF) and intravenous immunoglobulin (IVIg) were also used to deplete the complement or inhibit complement activation in experimental studies. Although the results were optimistic, few commercial products reached the market [10-12]. In addition, emerging evidence showed that complement was essential for tissue repair and regeneration [13]. Nevertheless, the role of complement components in cerebral ischemia is still poorly understood [14]. In this review, we will highlight recent discoveries about the roles of complement components in the pathophysiology of ischemic stroke and review the therapeutic strategies targeting complement both in clinical and experimental studies for ischemic stroke therapy. We aim to provide valuable information for exploring the role of the complement system and designing therapeutic strategies for ischemic stroke therapy in the future.

## 2. Complement in ischemic stroke

The complement system is essential for the innate immune response and plays a vital role in host defense and tissue homeostasis [15, 16]. The complement system is composed of more than 50 different plasma and membrane-associated proteins, and it can be activated through three different pathways: the classical, lectin, and alternative pathways [17]. Traditionally, it was considered that the complement system was primarily tagging and eliminating microbial intruders after activation. However, except for microbe elimination, the functions of the complement system have been highly extended over the past decades. To date, the complement system has been recognized as a participant in diverse processes, including clearance of immune complexes, mobilization of hematopoietic stem/progenitor cells (HSPC), angiogenesis, synapse pruning and maturation, tissue regeneration, and lipid metabolism [18]. Growing evidence has shown that the activity of the complement system is complicated and is involved in multiple immune, inflammatory, neurodegenerative, age-related, and ischemic diseases. In recent years, clinical observations and experimental studies have indicated that the complement system is crucial in the propagation of ischemic stroke [6, 19]. After cerebral ischemia, complement components synthesized by local activated cerebral endothelial cells, neurons, and glial cells, as well as complement derived from leukocytes, were strongly implicated in the progression of the disease [10].

### 2.1. C1q

C1q is well known to form the C1 macromolecule complex and initiates the classical complement pathway [20]. C1q contributes to the removal of infectious agents, apoptotic cells, and immune complexes [21]. Normally, C1q is present with proenzymes C1r and C1s in blood. However, C1q could also be synthesized in the brain after injury, such as viral infection, kainic acid treatment, and ischemic stroke [22-24]. In the developing visual system, C1q is expressed in synaptic regions of neurons and regulated by TGF-β. Depletion of C1q in knock-out mice resulted in impaired elimination of redundant synapses and excessive synaptic connectivity, which could lead to epileptogenesis and epilepsy [25-27]. In a normal brain, C1q is present in the neuropil, microglia, and a subset of interneurons [28]. After ischemic stroke, ischemic neurons predominantly expressed C1q, which may favor the attack or clearance of damaged neurons or cellular debris [24, 29]. In a model of transient middle cerebral artery occlusion (MCAO) in mice, salidroside treatment reduced C1q expression in the brain after 48 hours of brain ischemia, which was associated with enhanced NeuN and the expression of the growth response proteins Egr1, Egr2 and Egr4 [30]. In a model of hypoxia-ischemia in neonatal mice, selective depletion of C1q via the gene knock-out method reduced neutrophil infiltration, oxidative injury, and brain infarct volume after 24 and 72 hours of cerebral ischemia [31, 32]. These studies indicated that inhibiting C1q expression during the acute phase of cerebral ischemia could be beneficial to attenuate ischemic brain injury. However, another study showed that C1q knockout in adult mice subjected to MCAO surgery did not affect brain infarct volume at 24 hours after ischemia [33, 34]. Although the models of cerebral ischemia are different among the studies mentioned above, the pathological process of cerebral ischemia is common. Therefore, the authors speculated that the discordant results may result from the distinct maturation of the complement system between neonatal and adult mice [31]. Thus, the function of C1q and the effect of its upregulation on brain injury or tissue repair at different time points of cerebral ischemia need to be further investigated, especially in the late phase of the disease. These findings will help improve the design of proper therapeutic interventions targeting C1q against ischemic brain injury.

### 2.2. C3

#### 2.2.1. Introduction of C3

C3 is the most abundant complement protein in blood and is a central protein of all complement pathways [35]. Therefore, as one of the most extensively studied complement components, its structure and physiological function have been thoroughly studied [36-40]. C3 is activated and cleaved by C3 convertase into C3a and C3b. C3a could attract immune cells, modulate the immune response, and mediate downstream inflammatory responses by interacting with the cellular receptor complement component 3a receptor (C3aR). C3b and its degradation products iC3b and C3dg interact with cellular receptors, including complement receptor 1 (CR1, CD35), complement receptor 2 (CR2, CD21), complement receptor 3 (CR3, CD11b/CD18), complement receptor 4 (CR4, CD11c/CD18), and 'V-set immunoglobulin-domain-containing 4' (VSIg4, CRIg) on effector cells, mediating the clearance of pathogens and dying cells and modulating the adaptive immune response [41-45].

#### 2.2.2. C3 and ischemic stroke

##### Clinical studies of C3 in ischemic stroke

Clinical studies found that the level of C3 in plasma was higher in ischemic stroke patients than that in the healthy controls, and it peaked 3 days after brain ischemia [46-48]. Elevated C3 in plasma from embolic ischemic stroke or cryptogenic stroke patients was associated with worse neurological outcomes 3 months and 2 years after ischemia onset [49, 50]. Similarly, a recent study focusing on young ischemic stroke patients (18-50 years) also showed that the level of C3 in plasma was related to the prognosis 3 months after ischemic stroke [51]. In addition, genetic variation in the C3 gene was found to be associated with ischemic stroke, particularly with cryptogenic stroke [52]. These findings suggested that the systemic level of C3 could be a potential predictor of outcome after ischemic stroke.

##### Experimental studies of C3 in ischemic stroke

In the CNS, C3 has a role in nonimmune neuronal function [53-55] and is produced by several cell types, including neurons, astrocytes, microglia and oligodendrocytes [56-60]. It was reported that C3 was activated in ischemic tissue and implicated in ischemia-reperfusion injury [5, 61, 62]. C3 was increased in the brain after ischemic stroke, and inhibiting C3 activity could attenuate ischemic brain injury. In neonatal and adult rats subjected to hypoxia-ischemia surgery, the administration of CVF inhibited ischemia-induced C3 upregulation and reduced the brain infarct volume after 2 to 5 days of brain ischemia [63, 64]. In a mouse model of transient MCAO, inhibiting C3 activity via genetic knockout or antioxidant N-tert-butyl-α-phenylnitrone treatment could alleviate inflammatory response, reduce brain infarct volume, and attenuate neurological deficiency following 1 to 7 days of brain ischemia. *In vitro* studies showed that inhibiting C3 expression via small interfering RNA transfection enhanced cultured neuron viability under oxygen-glucose deprivation (OGD) conditions [34, 65]*.* These studies indicated that inhibiting C3 activity could attenuate ischemic brain injury, at least in the acute phase of cerebral ischemia. However, the role of C3 in the pathophysiology and tissue regeneration after ischemic stroke needs to be further investigated, especially in the subacute and chronic phases of stroke. In a mouse model of transient ischemic stroke, C3 deficiency via the genetic knock-out method reduced brain infarct volume and neutrophil influx following 1 day of ischemic stroke but impaired subsequent neurogenesis after 7 to 28 days of brain ischemia [5, 66-68]. These results implied that C3 was neuroprotective during the recovery process of cerebral ischemia, partially through promoting neurogenesis. Thus, C3 has a dual role during the pathogenesis of ischemic stroke. Therefore, C3 activity should be modulated rather than indiscriminately eliminated, such as with genetic knock-out or depletion with CVF, to attenuate C3-mediated injury after cerebral ischemia.

##### Experimental studies of C3a in ischemic stroke

C3a is a 9-kDa anaphylatoxic peptide derived from C3 and exerts its functions by binding to a specific receptor, C3aR, on effector cells [69]. C3a is involved in mediating both pro-inflammatory and anti-inflammatory activities after complement activation [70]. The pro-inflammatory activities include recruiting and activating leukocytes, promoting mast cell degranulation and smooth muscle contraction, increasing vascular permeability and vasodilation [71], and stimulating the synthesis of cytokines/chemokines [72, 73]. C3a-mediated inflammation contributed to the pathogenesis of pathological conditions in the CNS, such as experimental autoimmune encephalomyelitis [74] and ischemic stroke [34]. However, C3a was found to mediate the anti-inflammatory response and exert neuroprotective effects in a model of shock [75, 76]. *In vitro* studies showed that C3a treatment could protect neurons against excitotoxicity, stimulate microglia to produce neurotrophic growth factor, and promote the differentiation and migration of neural progenitor cells [77-80]. C3a treatment could also prevent cultured astrocytes from death induced by chemical ischemia or OGD by reducing ERK signaling and caspase-3 activation [81]. In a mouse model of neonatal hypoxia-ischemia, the intraventricular or intranasal administration of C3a 1 hour after ischemia ameliorated cognitive impairment after 6 weeks of cerebral ischemia. The intranasal administration of C3a did not affect tissue loss or the expression of synaptic plasticity-related proteins of pansynaptic marker synapsin I and growth-associated protein 43 in hippocampus [82, 83]. In an adult mouse model of photothrombotic stroke, the intranasal administration of C3a after 7 days of brain ischemia enhanced pansynaptic marker synapsin I and growth-associated protein 43 expression in the peri-infarct cortex and accelerated the complete recovery of forepaw motor function [84]. Although these studies used different models of cerebral ischemia in mice of different ages, the results suggested that administration of C3a at a certain time after cerebral ischemia was beneficial for neurological function recovery. Since the intranasal administration of C3a in neonatal and adult mice after cerebral ischemia resulted in different expression levels of synaptic plasticity-related proteins in the brain, future studies aiming to explore the therapeutic mechanism of C3a on promoting synaptic plasticity need to consider both age and animal model type. In addition, the optimal delivery route of C3a administration for ischemic stroke therapy also needs to be further studied.

##### Experimental studies of C3b and iC3b in ischemic stroke

C3b is another activated fragment of C3 and can be further cleaved into the inactive end fragment of iC3b after recognizing and binding several complement regulators, including factor H (FH), decay accelerating factor (DAF, CD55), and membrane cofactor protein (MCP, CD46) [85-89]. The complement regulators regulate the generation of C3b and stop C3b opsonization by breaking down the C3 convertases, protecting host cells and tissues from inadvertent complement activation. Dysfunction of these regulatory proteins can be due to familial mutations in the complement genes or the presence of autoantibodies against regulators linked to atypical hemolytic uremic syndrome (aHUS), C3 glomerulopathies (C3G), dense deposit disease (DDD) in kidneys and age-related macular degeneration (AMD) in eyes [90-94]. C3b and iC3b could mediate significant signaling functions and induce opsonophagocytosis of targeted cells and particles through interacting with the complement receptors CR1, CR2, and CR3, which are critical in host defense and homeostasis [15, 16, 95-98]. It was reported that apoptotic cells could induce complement activation and the generation of C3b/iC3b [99, 100]. C3b/iC3b was deposited on apoptotic and necrotic cells and apoptotic bodies, which facilitated the phagocytosis and removal of the cells or particles by macrophages expressing the receptors CR1 and CR3 [101, 102]. In a mouse model of CCL4-induced liver injury, C3b/iC3b was deposited in damaged liver parenchyma. C3b/iC3b deficiency caused by genetic knock-out C3 or complement depletion via CVF treatment led to a slow removal of damaged tissue and impaired regeneration after 72 hours of injury [103]. In a mouse model of *Clostridium difficile* -induced intestinal damage, C3b exerted the function of opsonization and promoted the phagocytosis of bacteria by neutrophils [104]. These results suggested that the involvement of C3b/iC3b in the elimination of pathogens or injured tissue contributed to tissue repair and recovery after injury. Apart from mediating phagocytosis under pathological conditions, C3b/iC3b was beneficial for embryo development. Oviductal cell-derived C3b/iC3b enhanced the size of blastocysts and the rate of hatching, promoting embryo development and reproduction in mice [105]. In human and mouse multiple sclerosis, deposited C3b/iC3b was implicated in microglia priming and in the acceleration of the progression of the disease [106]. These findings suggest that blocking the interaction of C3b/iC3b with microglia may be a unique therapeutic approach to neurodegenerative disease. However, to date, the role of C3b/iC3b in ischemic stroke is largely unknown. Studies focusing on the relationship between C3b/iC3b-mediated phagocytosis and the outcomes in ischemic stroke are also scarce.

### 2.3. CR1 in ischemic stroke

CR1 is a single-chain membrane-bound glycoprotein expressed predominantly on blood cell types, of which >80% of CR1 is dedicated to erythrocytes [107-110]. It is a receptor for C3b and C4b [111]. In the periphery, CR1 is known to play several pivotal roles; one important function is capturing and clearing C3b/C4b-opsonized pathogens or immune complexes via their transport to the liver or spleen [111, 112]. Failure to clear the immune complex leads to the deposition of complexes in tissues and activation via Fc receptors, resulting in tissue injury. CR1 expressed on certain leukocytes could also promote phagocytosis of complement-opsonized cells [113].

The expression and distribution of CR1 in humans and rodents is different because human CR1 is encoded by a separate gene to human CR2, while murine CR2 encodes both CR1 and CR2 [114-116]. In the CNS of humans, the function of CR1 has not been well established because even the presence of CR1 in the human brain is limited and conflicted [115, 117-120]. Recent studies showed that CR1 mRNA could be detected in the cortex and cerebellum of Alzheimer's disease (AD) patients but in very low amounts [121-123]. Recent Genome Wide Association Studies (GWAS) have identified that CR1 is strongly implicated in the progression of AD [124-128]. An *in vitro* study showed that CR1 expressed on microglia was increased after microglia activation induced by lipopolysaccharide (LPS). The antibody blockage of CR1 enhanced microglial phagocytosis of dextran beads under Amyloid-β 42 treatment. In addition, the blockage of CR1 on microglia following LPS or amyloid-β 42 treatment prevented neuronal death induced by conditioned medium from LPS- or Amyloid-β 42-stimulated microglia [129]. This study suggested that microglial CR1 was detrimental to neurons in the pathology of AD. However, in the pathological process of ischemic stroke, the role of CR1 is still unknown. More studies need to be performed in the future to elucidate the function of CR1 in ischemic stroke.

### 2.4. CR2 and sCR2 in ischemic stroke

In the immune system, CR2 (CD21) is primarily expressed on B cells, dendritic cells, and a subset of T cells [113, 116, 130, 131]. CR2 is a key part of the B cell antigen receptor complex together with CD19, CD81, and CD225. It has been known to result in a more efficient humoral immune response via binding to its ligands, mainly including the breakdown products of C3b (iC3b, C3dg, and C3d) and antigen-bound IgM [132]. CR2 was also found to play a role in B cell differentiation, selection, maintenance and elimination of self-reactive B cells [133]. Variations or deletions of the CR2 gene in humans or in mice are associated with a variety of autoimmune and inflammatory conditions, such as systemic lupus erythematosus (SLE) and chronic variable immunodeficiency (CVID) [116]. However, in the CNS, the function of CR2 is still unclear. Previously, the expression of CR2 was found in human glioma cells and fetal astrocytes, but its expression in mouse brains was much lower [117, 134]. Recently, CR2 was shown to be expressed in neural progenitor cells and to regulate hippocampal neurogenesis. A lack of CR2 in young and old CR2 knock-out mice increased basal neurogenesis compared with wildtype littermates, while the intracerebral injection of the CR2 ligand of C3d reduced the proliferating neuroblasts in wildtype mice but not in CR2 knock-out mice [134]. In another study, a genetic depletion of CR2 in mice improved neurological outcomes and reduced mortality after traumatic brain injury. In addition, deficiency of the CR2 gene also attenuated C3 and IgM deposition in the brain, as well as inhibited astrocytosis and microglial activation at 7 days post-injury [135]. In a mouse model of spinal cord injury, CR2 was upregulated in the spinal cord after 7 days of injury. CR2 deficiency led to an increased loss of synaptic nerve terminals following nerve injury. An *in vitro* study showed that astrocytes were the main cell type expressing CR2 [136]. These studies suggested that CR2 plays a functional role in the response to tissue injury, as least in a mouse model of closed head injury and spinal cord injury.

In regard to ischemic stroke, the role of CR2 in the brain has been scarcely investigated until now. However, it has largely been known as a potential therapeutic vehicle by linking complement-inhibitory proteins, such as Crry, fH, and CD59, to inhibit complement activation in various diseases, such as collagen antibody-induced arthritis, ischemia reperfusion injury, acute lung ischemia reperfusion injury, cardiac ischemia reperfusion injury, and cerebral ischemic injury [5, 61, 130, 137-141]. Crry inhibits all complement pathways at the C3 activation step, fH inhibits only the alternative pathway, and CD59 inhibits generation of the terminal membrane attack complex (MAC, C5b-9) [61, 142, 143]. Therefore, the different fusion variations of the complement inhibitors, namely, CR2-Crry, CR2-fH, and CR2-CD59, make it possible to determine the role and contribution of certain complement pathways and activation products in disease processes.

In a mouse model of 60 min transient cerebral ischemia, a treatment of CR2-Crry via tail injection improved neurological outcomes, reduced neutrophil influx in the brain, and increased blood flow after reperfusion [68]. In the same model in mice, another study investigated the effect of either CR2-Crry or CR2-fH treatment on outcomes after ischemic stroke. The results showed that CR2-Crry treatment reduced brain infarct volume and neurological deficit after ischemia but did not show a protective effect after ischemia. Furthermore, CR2-fH treatment displayed persistent neuroprotective function, as both reduced ischemic brain injury and improved neurological function after ischemia were observed [5]. In fact, a previous study also found that CR2-fH treatment mediated protection from cerebral injury until 7 days post-reperfusion in mice [138]. Thus, the alternative pathway of complement activation possibly played a detrimental role for a relatively longer period during the pathophysiological process of ischemic stroke. Inhibiting all the pathways of complement activation via CR2-Crry treatment is not the best option for reducing complement-related ischemic brain injury because it affects the subsequent tissue regeneration after ischemic stroke. The role of CR2-CD59 has been reported in a mouse model of choroidal neovascularization (CNV), where it contributes to emolliating the severity of choroidal neovascularization [144]. However, the effectiveness of CR2-CD59 treatment for ischemic stroke has not yet been clarified.

The soluble form of CR2, sCR2, has been demonstrated to shed from human B cells, T cells, and lymphocytes and is present in the circulation [145-148]. Decreased sCR2 levels in the circulation were shown in multiple autoimmune diseases, such as multiple sclerosis, systemic sclerosis, and rheumatoid arthritis [149, 150]. A recent study found that the level of sCR2 was elevated in the cerebrospinal fluid of multiple sclerosis patients and correlated with the severity of the disease. Furthermore, an *in vitro* study showed that sCR2 inhibited the cleavage of C3 into iC3b, which mediated the clearance of debris from the inflammatory site [151-153]. These findings suggest a novel function of sCR2 in human neuro-inflammation. In the pathogenesis of ischemic stroke, the change and function of sCR2 is unclear and warrants comprehensive investigation in the future to discover new targets for the treatment of ischemic stroke.

### 2.5. C3aR in ischemic stroke

C3aR is the receptor of C3a and belongs to the rhodopsin-family of seven transmembrane domain G-protein-coupled receptors [154, 155]. C3aR is expressed not only in cells of the myeloid lineage (monocytes, macrophages, eosinophils, basophils) but also in many tissues and cell types outside of the immune system [39, 156-158]. There are several differences in the C3aR expression patterns between humans and rodents. Human neutrophils, mast cells, and endothelial cells express C3aR, while no data support the C3aR expression in these cells in mice [159, 160]. In addition, various stem and progenitor cells were shown to express C3aR, including hematopoietic stem cells, mesenchymal stem cells, neural stem cells, and dental pulp progenitor cells [67, 161-163]*.* One study reported that C3aR was necessary for basal neurogenesis. Mice lacking C3aR or mice treated with a C3aR antagonist reduced DCX^+^/BrdU^+^ cells in the subventricular zone (SVZ), the dentate gyrus subgranular zone (SGZ) of the hippocampus, and the olfactory bulb (OB) [67].

In the CNS, microglia, astrocytes, oligodendrocytes, and neurons express C3aR, among which the neurons were identified as the principle cell type expressing C3aR under physiological conditions [164-167]. It has been shown that C3aR is involved in chick eye morphogenesis and rat cerebellar cortex histogenesis during development and facilitates mouse skeletal muscle regeneration after cardiotoxin-induced muscle injury [168-170]. However, in multiple CNS diseases, C3aR expression was increased in the brain, contributing to the exacerbation of inflammation and disease progression. For example, C3aR was implicated in the pathophysiology of major depressive disorder (MDD) [171]. C3aR expression increased in the prefrontal cortex of depressed suicide specimens and in mice with stress-induced depressive-like behavior. C3aR genetic knock-out inhibited C3aR^+^ monocyte infiltration into the brain and reduced the levels of the pro-inflammatory cytokine IL-1β in the prefrontal cortex after chronic stress. In addition, deficiency of C3aR prevented chronic stress-induced behavior despair. In a mouse model of AD, C3 and C3aR expression levels were upregulated and specifically expressed in astrocytes and microglia in the brain, respectively [172]. Depletion of C3aR through genetic knock-out or C3aR antagonist treatment in microglia reversed the ability of microglia phagocytosis under chronic C3 stress. C3aR antagonist treatment in AD mice reduced plaque load and microgliosis in the brain. The study indicated that microglial C3aR together with C3 mediated β-amyloid pathology and neuro-inflammation in an AD mouse model [172]. In regard to ischemic brain injury, it was reported that C3aR was expressed on endothelial cells, microphage-like cells, and astrocytes, displaying upregulation after 6 hours to 2 days of focal ischemia in mice [173]. Using a mouse transient or permanent MCAO model, C3aR was implicated in the recruitment of neutrophils to the infarct zone and exacerbated tissue injury; however, this effect depended on blood reperfusion, as the protective effect of intraperitoneal C3aR antagonist on reducing brain infarct volume and inflammatory cell infiltration in the brain was not observed in animals after permanent MCAO [174]. In contrast, in a neonatal rat model of hypoxic-ischemic brain injury, the intranasal or lateral cerebral ventricle injection of C3a 1 hour after brain injury could attenuate tissue loss and memory impairment 41 days after brain injury in wild-type mice compared to C3aR genetic knock-out mice [82, 83]. These studies suggested that C3a/C3aR exerted a protective role in the pathogenesis of hypoxic-ischemic brain injury in neonatal rats. One of the reasons causing the conflicting results of the role of C3aR in brain ischemic injury could be the different pathological process between transient MCAO and hypoxic-ischemic brain injury. The other reason could be the different susceptibility of cerebral ischemia in adult and neonatal animals. In addition, all studies mentioned above mainly focused on the role of C3aR in the acute phase of cerebral ischemia, but the function of C3aR in the subacute and chronic phases of ischemic stroke is still unknown and needs to be further investigated.

In addition to its role in ischemic stroke, C3aR was also reported to be implicated in the progression of thoracic aortic dissection and myocardial infarction, which are also vascular diseases [175, 176]. In a model of brain inflammation induced by LPS injection, the results showed that C3aR played a critical role in endothelial activation and leukocyte recruitment when LPS was injected into the brain [177]. In a mouse model of laser-induced macular degeneration, the presence of C3aR was associated with increased detrimental angiogenesis in the retina [178]. This study showed that C3aR has a close link with the function of endothelial cells and angiogenesis under pathological conditions. However, whether C3aR affects the function of endothelial cells and angiogenesis after ischemic stroke remains unclear. The dysfunction of endothelial cells could result in a variety of severe events, such as the impairment of the integrity of the blood brain barrier (BBB) and angiogenesis, both of which play an important role in post-stroke recovery. Therefore, investigating the role of C3aR on the integrity of BBB and angiogenesis after ischemic brain injury is also warranted and would be helpful for uncovering the novel function of C3aR during ischemic stroke.

### 2.6. CR3 in ischemic stroke

#### 2.6.1. Introduction of CR3

CR3, also called αMβ2, CD11b/CD18, or Mac-1, belongs to the integrin β2 subfamily. CR3 is functionally composed of CD11b (α-chain) and CD18 (β-chain) [179]. It was noted that CD11b is the key subunit mediating the biological functions of CR3 and is encoded by the ITGAM gene in humans, while CD18 is a common subunit shared with the other three members of the integrin β2 subfamily, including CD11a/CD18, gp150/95 (CD11c/CD18), and CD11d/CD18 [179, 180]. CR3 is highly expressed in most myeloid and lymphoid cells in both humans and rodents [181]. CR3 exhibits broad ligand recognition specificity and has more than 40 reported protein ligands, which explains the complexity of CR3-mediated functions [182, 183]. It is widely accepted that CR3 mediates the adhesive reaction of leukocytes during the inflammatory response and particularly promotes the migration of neutrophils to sites of inflammation [184-186]. CR3, especially the subunit CD11b, mediates the mobility of neutrophils, which is beneficial for controlling infection [187]. CR3 was also shown to suppress inflammation by affecting pro-inflammatory signaling pathways, such as the toll-like receptor (TLR) and FcγR signaling pathways [188-191]. For example, CD11b on leukocytes was rapidly activated after TLR stimulation [180, 192, 193]. Activated CD11b on macrophages induced the intracellular activation of Src and Syk, which phosphorylated MyD88 and TRIF and eventually led to reduced activation of the transcription factor NF-κB and the production of pro-inflammatory proteins. By contrast, a deficiency of CD11b in macrophages reversed this phenomenon [189]. The inhibition of the production of pro-inflammatory cytokines was also observed on NK cells pretreated with CR3-specific agonists under TLR stimulation [191]. These studies suggested that CR3 could negatively regulate inflammation induced by TLR-dependent pathway activation.

Another CR3 function is enhancing phagocytosis, which is also the main role of CR3 [181]. CR3 was first demonstrated to induce phagocytosis via the interaction between the subunit CD11b and its ligand iC3b. These activated fragments exist during complement pathways, and is the reason why CR3 is named as a complement factor [194, 195]. Under pathophysiological conditions, cells expressing CR3 could phagocytize iC3b opsonized bacteria, apoptotic cells and immune complexes, which was essential in limiting the unwanted inflammatory immune response [196-199]. In addition, the interaction between CR3 and iC3b also contributed to the release of anti-inflammatory cytokines such as IL-10 and TGF-β [200]. CR3 was also involved in the pathogenesis of systemic lupus erythematosus. A single nucleotide polymorphism in the CD11b chain (rs1143679) could impair CR3-mediated phagocytosis in monocytes and monocyte-derived macrophages isolated from patients with this mutation [201, 202].

#### 2.6.2. The role of CR3 in the CNS

Numerous studies have demonstrated that CR3-mediated phagocytosis is critical during CNS development, for the homeostasis of the microenvironment in the brain, and for repair and regeneration after injury [25, 203-206]. CR3 was spatially expressed next to synapses and played a crucial role in synaptic pruning and plasticity during brain development [115, 207, 208]. In the CNS, microglia were the only cell type expressing CR3 and exerted a phagocytosis function under pathophysiological conditions [115, 207, 209, 210]. In the postnatal retinogeniculate system, microglia phagocytized presynaptic inputs during peak synaptic pruning, which depended on neuronal activity and the CR3/C3 phagocytic signaling pathway. Blocking the interaction between microglia-specific CR3 and C3 led to sustained deficits in synaptic connectivity and the removal of extranumerary synaptic inputs [211, 212]. This study further supported the significant role of microglia-specific CR3 in synaptic remodeling during development. However, the CR3-mediated phagocytosis of synapses was involved in the pathogenesis of AD in animal experiments. AD mice lacking CR3 showed less phagocytic synapses in microglia and improved synaptic function in the brain. Inhibiting CR3 activity could reverse synaptic loss and dysfunction during the pathology of AD [213]. Deficiency of the CR3-related adapter protein in AD mice could attenuate the severity of neurotic dystrophy and learning deficits [214]. In addition, clinical studies found that the mutation of microglia-specific receptors such as TREM2, CD33, and CR3 were associated with increased risks for developing AD [215]. Thus, therapeutic targeting of CR3 and microglia activation at the early stage may reduce synaptic loss and hinder the progression of AD. Despite the detrimental effect of CR3-mediated phagocytosis on synapses, it was also found that CR3 could uptake fibrillary amyloid-β (fAβ) in microglia both *in vitro* and *in vivo* , which contributed to the clearance of Aβ during AD progression [216]. By contrast, a recent study uncovered a distinct mechanism of microglia-specific CR3 on modulating the Aβ levels in the brain [217]. In a mouse model of AD, the genetic knock-out of CR3 caused increased Aβ accumulation in the brain compared to the wide-type mice. The *in vitro* study also showed that microglia with CR3 depletion displayed higher efficiency at degrading extracellular Aβ by secreting enzymatic factors than that of wild-type cells [217]. This study provides novel insight into the CR3- and microglia-mediated removal of brain Aβ in AD, which is independent of phagocytosis.

#### 2.6.3. The role of CR3 in ischemic stroke

CR3 could be directly activated under a variety of neuro-inflammatory stimulations [218]. After ischemic stroke, CD11b, the subunit of CR3, was upregulated in the brain and was commonly recognized as a marker for microglia activation [219-221]. CD11b was also used to define bone marrow derived macrophages and CD11b^+^ macrophages were involved in impairing neovascularization in ischemic muscle in aged mice [222]. Generally, CR3-mediated phagocytosis was beneficial for clearing debris, apoptotic or necrotic neurons, immature synapses, or degenerated tissue and limiting inflammation after injury [25, 179, 207, 223, 224]. However, the precise function of CR3 and its mediated phagocytosis was not completely investigated during cerebral ischemia. Several studies used mice with the genetic depletion of CD11b or CR3 to explore the role of CR3 in the pathological process of ischemic stroke, but it is still controversial whether CR3 exerts protective or detrimental effects after cerebral ischemia. Using CD11b-thymidine kinase mutant-30 (TK^mt-30^) transgenic mice, in which CD11b^+^ microglia were depleted in the brain after ganciclovir injection, a lack of CD11b^+^ microglia resulted in increased apoptotic neurons and a larger infarct volume at 3 days after ischemia [225]. The results revealed a neuroprotective role of CD11b during ischemic stroke. However, the beneficial role of CR3 in the acute phase of ischemic stroke was challenged by another study in which anfibatide, the platelet glycoprotein (GP) receptor Ibα inhibitor, was used to treat ischemic stroke. The results showed that anfibatide injection 1-hour post-ischemic reperfusion inhibited the elevation of CR3 expression. This phenomenon was associated with reduced BBB damage, smaller brain infarct volume, and improved neurological functions after 24 hours of ischemia [226]. The detrimental role of CR3 in stroke-induced BBB damage and brain injury was supported by other studies. In a thrombotic stroke mouse model, CR3 deficiency protected from intracerebral hemorrhage induced by thrombolysis with tissue plasminogen activator (tPA) 5 hours after MCAO [227]. In a model of 90 min or 180 min MCAO, mice lacking CR3 showed less BBB permeability, less neutrophil infiltration, and reduced infarct volume after 24 hours of ischemia compared to wild-type mice [227, 228]. The intravenous injection of CD11b or CD18 monoclonal antibodies in rats at 2-4 hours after transient cerebral ischemia also showed protective effects after 2 days and 7 days of cerebral ischemia [229, 230]. Although these studies indicated that CR3 possibly acted as a deleterious factor for ischemic brain injury, it is still difficult to define the exact role of CR3 in the pathogenesis of cerebral ischemia, especially at the late stage of ischemic stroke. Although an antagonist of CR3, XVA143, has been used to curb CR3 activity in some studies, it is not available from the market, and no specific inhibitors for CR3 are available up to now [231-234]. This further increases the difficulty of studying the role of CR3 under pathological conditions. It was found that CD11b could be activated and sustained upregulation for a long time after cerebral ischemia; however, the study of the function of CD11b at the late phase of ischemic stroke is poorly understood [219]. In addition, although much evidence has shown microglia-specific CR3-mediated synaptic remodeling or loss during development or in a variety of diseases, less is known about the relationship between CR3 activation and synaptic remodeling or loss in ischemic stroke. Further work focusing on the area will broaden our knowledge of CR3 in ischemic stroke. UK-279, 276, also referred to as recombinant neutrophil inhibitory factor, has been called a selective CR3 antagonist. It was found to bind to the I-domain of CD11b and inhibited neutrophil adhesion [235]. It has been shown to be helpful for attenuating acute ischemic brain injury at the early stage of stroke [236-238]. Therefore, it may be a potential tool for exploring CR3-mediated functions during the late phase of ischemic stroke.

### 2.7. C5, C5a, and C5b-9 in ischemic stroke

#### 2.7.1. Introduction of C5, C5a and C5b-9

C5 is considered to be at the center of complement activation and exerts biological activities after being cleaved into C5a and C5b by either the classic or alternative pathway C5 convertases [239]. The C5 convertases (C4b2a3b and C3b2BbP) cleave C5 to a smaller active fragment of soluble complement factor 5a (C5a) and another larger fragment of membrane-bound complement factor 5b (C5b) [13, 240]. Similar to C3a and C4a, C5a is also termed anaphylatoxin. C5a consists of 74 amino acids and induces the release of various mediators from mast cells and phagocytes, which in turn amplify inflammatory responses [240, 241]. C5a acts as a chemoattractant for cells such as neutrophils and monocytes to the sites of injury or inflammation and can increase the production of interferon gamma (IFN-r) [242]. Compared to C3a, C5a has a higher potency and obviously more biological action [243, 244]; it exerts multiple functions via binding to its receptors complement component 5a receptor 1 (C5aR1/C5aR/CD88) and complement component 5a receptor 2 (C5aR2/C5L2/GPR77) [10, 113, 245, 246]. Through the interaction with C5aR1 on polymorphonuclear leukocytes (PMNs), monocytes, and macrophages, C5a could induce the secretion of lysosomal enzymes and pro-inflammatory cytokines [247]. When binding to C5aR1 on neutrophils, C5a enhanced the adhesiveness and aggregation of neutrophils and promoted oxidative metabolism and the production of reactive oxygen species (ROS) in neutrophils [248, 249]. Unlike the interaction between C5a and C5aR1, the interaction between C5a and C5aR2 did not induce classical signaling (induction of intracellular calcium transients) or cause biological cellular responses [250]. The precise biological function of C5aR2 remains unclear [113, 251-254]. C5aR2 appeared to be a non-signaling or potential decoy receptor for C5a [246, 255]. However, recent studies found that C5aR2 acts as a functional receptor in sepsis, as C5aR2 hindered the release of high mobility group box 1 (HMGB1) protein from macrophages; moreover, the blockage of C5aR2 along with C5aR1 improved survival in sepsis compared to the blockage of either receptor alone [256]. Thus, the role of C5aR2 still awaits comprehensive investigation in the future [246].

C5b-9 is the membrane attack complex (MAC), which is also called the terminal complement complex (TCC) and is formed by C5b subsequently binding to complement factor 6 (C6), complement factor 7 (C7), complement factor 8 (C8) and complement factor 9 (C9). C5b-9 on the target surface could lead to cell lysis or cell activation, depending on the level of C5b-9 deposition on the cell surface [96, 113, 257-259]. The combined functions of C5a and C5b-9 are strongly responsible for the inflammation and tissue damage associated with excessive complement activation [10].

##### Clinical studies of C5, C5a and C5b-9 in ischemic stroke

A variety of genetic polymorphisms in complement genes have been shown to be linked to atherosclerosis, cardiovascular disease, and ischemic stroke [260, 261]. Among numerous genes, a polymorphism in the C5 gene (C5 rs17611) was the only independent risk factor for transient ischemic attack (TIA) or stroke and was associated with an increased incidence of stroke in patients with carotid atherosclerosis [262-264]. In addition, the polymorphism in the C5 gene was also related to higher plasma C5a levels and predicted increased incidence of stroke in patients with carotid atherosclerosis [262, 263].

In patients with acute ischemic stroke, both C5a and C5b-9 changed in the plasma. A study of 15 acute ischemic stroke patients demonstrated that C5a in the plasma began to increase at 7 days and was maintained at a plateau up to 14 days, while C5b-9 decreased after 1 and 2 days of ischemic stroke and showed no difference up to 28 days after ischemic stroke compared to the healthy control [48]. By contrast, another study recruiting 11 acute ischemic stroke patients found that, compared to the healthy control, the plasma C5b-9 was elevated at 72 hours, peaked at the 7^th^ day, and maintained at a plateau up to 12 days, which correlated to the infarct volume within a follow-up period of 12 months [265]. Utilizing severe carotid atherosclerosis as the control, a study recruiting 26 ischemic stroke patients did not find changes in plasma C5a. However, the level of plasma C5b-9 was significantly elevated and exhibited a positive correlation with the clinical severity of ischemic stroke and the level of functional disability [266]. Although these studies presented discrepant results regarding the changes in plasma C5a and C5b-9 in acute ischemic stroke, which may be due to the distinct severity of patients or the detection methods, it is no doubt that C5a and C5b-9 are activated during stroke pathophysiology. Studies recruiting a large scale of different subtypes of stroke are imperative to confirm the changes in plasma C5a and C5b-9 in ischemic stroke and their correlation with the outcomes of stroke. These findings pave the foundation for the use of C5a and C5b-9 in the plasma as a predictor for the prognosis of ischemic stroke.

##### C5/C5a in experimental ischemic stroke

C5 occupies a critical position within the complement cascade, as it could be generated through all three complement pathways [267]. The role of C5, even at the early stage of ischemic brain injury, remains unclear [268]. In a mouse model of 60 min transient MCAO, C5 genetic knock-out did not offer neuroprotection after ischemia [34]. Conversely, in a mouse permanent MCAO model, C5 deficiency both reduced brain infarct volume and attenuated neurological deficits after ischemia [269]. One possible explanation for the opposite results between the two studies was the difference in brain blood reperfusion. Systemic C5 inhibition via intravenous and intraperitoneal injection of anti-C5 monoclonal antibodies also prevented the deterioration of neurological functions by reducing cerebral lesion and edema after ischemic brain injury [270]. The lifelong deficiency of C5 via genetic knock-out could result in a constitutionally detrimental effect since completely acute C5 blockade might mask a potential benefit in the context of stroke. Therefore, focal or temporal C5 inhibition during the peri-ischemic time period, instead of lifelong C5 deficiency, could be a better option for reducing the C5-mediated detrimental effects after ischemic stroke. Apart from acute ischemic brain injury, C5 was also activated and continued for a long time during the pathogenesis of chronic cerebral ischemia [271]. In a rat model of chronic cerebral hypoperfusion, C5 deposition in the corpus callosum was increased after 30 days of bilateral carotid artery stenosis. C5-deficient mice showed a decrease in white matter injury in the corpus callosum and fewer reactive astrocytes and microglia compared to the wild-type mice. This study focused on the role of C5 in chronic cerebral ischemia, illustrating the relationship between C5 deposition and chronic cerebral ischemia-induced white matter injury [271]. However, a direct causal role for C5 in the chronic phase of acute ischemic stroke was not assigned, which needs to be comprehensively investigated in the future.

The anaphylatoxin C5a, a split product of C5, is constantly produced during complement activation [272]. C5a is a major pro-inflammatory mediator in a wide range of diseases because it regulates both the inflammatory process in innate immunity and the adaptive immune response [273]. C5a affects inflammation by inducing the release of cytokines and chemokines, upregulating adhesion molecule expression, and increasing vascular permeability [274, 275]. Furthermore, C5a is able to activate endothelial cells and induce caspase-dependent endothelium apoptosis in experimental lupus [276-278]. Numerous studies have shown that the generation of C5a and its interaction with the receptor C5aR1 played a deleterious role in multiple diseases, including atherosclerosis, arthritis, renal ischemia-reperfusion injury, small intestine ischemia-reperfusion injury, mesenteric ischemia, myocardial ischemia, and ischemic stroke [272, 279-284]. In a mouse model of MCAO, C5a was upregulated after 1 day of ischemic stroke and predominantly generated by neurons in the brain [34, 285]. Blocking C5a signaling via genetic knock-out of C5aR1 in mice improved neurological scores and reduced infarct size after 1 day of ischemic stroke. An *in vitro* study confirmed that cultured neurons expressed and upregulated C5a expression under ischemic stress. Cultured astrocytes and microglia showed no elevation of C5a expression under ischemic conditions. C5a treatment induced neuronal apoptosis under OGD conditions, while C5aR1 deficiency prevented neurons from OGD-induced apoptosis, suggesting that inhibiting the interaction between C5a and C5aR1 was neuroprotective in the acute phase of ischemic stroke [285]. Despite the overall body of evidence indicated that C5a activation generated harmful effects during the early stage of ischemic stroke, C5a possessed neuroprotective potential against glutamate-mediated neurotoxicity in mice [286, 287]. Therefore, it is possible that C5a might act as a protective factor to inhibit tissue injury in the late phase of cerebral ischemia. Since the temporal course and functional significance of C5a during the progression of ischemic stroke have been extensively studied, the molecular mechanism of C5a in ischemic stroke needs to be explored in the future.

##### Experimental studies of C5b-9 in ischemic stroke

C5b-9 is an end product during the complement cascade. *In vitro* studies showed that a sublytic dose of C5b-9, which did not cause cell death, induced proto-oncogenes, activated the cell cycle, and increased cell survival in oligodendrocytes [288]. C5b-9 prevented cultured oligodendrocytes from apoptotic cell death by inhibiting caspase-3 and caspase-8 activation and Bid cleavage and increasing Bcl-2 expression and cellular FLIP expression [289, 290]. C5b-9 triggered the secretion of interleukin-8 (IL-8) and monocyte chemoattractant protein-1 (MCP-1) from human umbilical vein endothelial cells [291]. Erythrocyte-derived C5b-9 could induce the constriction of cultured rat cerebral artery smooth-muscle cells, indicating that C5b-9 could induce cerebral vasospasm [292]. These findings suggest that C5b-9 could play a role to some extent under pathophysiological conditions.

Indeed, *in vivo* animal studies proved that C5b-9 was implicated in the pathogenesis of various diseases, including kidney ischemia-reperfusion and transplantation, spinal cord injury, traumatic brain injury, multiple sclerosis, AD and stroke [288, 293-295]. The involvement of C5b-9 in diseases was further supported by the observations of its deposition in patients. For example, C5b-9 was deposited in some cerebral arterial walls in the cerebral amyloid angiopathy [288]. In multiple sclerosis patients, C5b-9 presented in the brain within the plaques close to endothelial cells and adjacent to white matter [296]. In AD patients, deposition of C5b was noticed in the lesion area, which was associated with dystrophic neurites, neurofibrillary tangles, and Aβ deposits [297, 298].

Although it is apparent that there exists a relationship between C5b-9 expression and the pathogenesis of CNS disease, a pathological role for C5b-9 in stroke has not been defined. Deposition of C9, a marker for C5b-9 assembly, was determined in neurons in the infant brain with hypoxic-ischemic encephalopathy [299]. In a hypoxia-ischemia model in neonatal rats, C5b-9 was neurotoxic because the deficiency of C9 reduced brain infarct volume after 24 hours of stroke, while C9 administration reversed the result [300]. In a mouse model of transient cerebral ischemia, deficiency of C6, a member of C5b-9, did not prevent brain injury after 24 hours of reperfusion. Similarly, utilizing the same model, genetic knock-out of CD59a (a molecule that can inhibit the generation of C5b-9 ) did not affect the outcomes after 72 hours of reperfusion [138, 301]. However, in a mouse model of MCAO, the deficiency of CD59a increased brain infarct volume and exacerbated neurological deficit after 72 hours of ischemia [301]. These studies indicated that the function of C5b-9 during ischemic stroke may be dependent on the type and severity of insult.

### 2.8. C5aR1 in ischemic stroke

#### 2.8.1. Introduction of C5aR1

C5aR1, known as CD88, is a 45 k-Da protein and belongs to the rhodopsin family of seven-transmembrane G protein-coupled receptors (GPCRs) [159, 302]. It is the primary receptor of C5a and plays a pivotal role in pro-inflammatory and regulatory functions [159, 302]. C5aR1 is mainly expressed in myeloid cells such as neutrophils, monocytes, macrophages, basophils and eosinophils [159]. Nonmyeloid cells, including NK cells, NKT cells, T cells, epithelial cells, endothelial cells, smooth muscle cells and neural cells, also express C5aR1 [303-310]. In both the human and mouse brain, C5aR1 is constitutively expressed in neurons and glial cells at a low level [54, 165, 311-317]. However, under inflammation or disease states, in both human and experimental animal models, C5aR1 expression was greatly upregulated in astrocytes, microglia, and to a lesser extent on endothelial cells [54, 173, 308]. Increased C5aR1 expression was reported in Huntington disease, allergic encephalomyelitis, pyogenic granulomas of human skin [318-320], the CNS during inflammation [166, 308, 321], amyotrophic lateral sclerosis [322], closed head injury [323], and ischemic stroke [173].

#### 2.8.2. The role of C5aR1 in brain development

Accumulated evidence has shown that C5aR1 is implicated in brain development. C5aR1 is transiently expressed on rat cerebellar granule neurons, with intense expression at 12 days but disappearance at 30 days after birth. An *in vitro* study confirmed C5aR1 expression in cerebellar granule neurons, and the expression was increased during neuronal differentiation and maturation. C5aR1 agonist treatment could prevent cultured cerebellar granule cells from serum deprivation-induced apoptosis, suggesting that C5aR1 could provide anti-apoptotic signaling to granule neurons during brain development [312]. The role of C5aR1 during development was further supported by another study. The injection of a C5aR1 agonist at the surface of the cerebellum promoted the proliferation of immature neurons and enlarged the thickness of the external granule cell layer (EGL) [168]. The contribution of C5aR1 to CNS development is not restricted to the cerebellum. For example, C5aR1 is expressed in presynaptic terminals of mossy fibers within the hippocampal CA3 region, suggesting a role for C5aR1 in synaptic/cellular plasticity [324]. More recent data supported the beneficial role of C5aR1 in neurogenesis in the brain [325, 326]. C5aR1 was expressed in cultured neural progenitor cells and in the subventricular zone of the brain [67, 327]. Blocking C5aR1 signaling via a selective C5aR1 antagonist PMX53 inhibited the proliferation of neural progenitor cells in the embryonic subventricular zone *in vivo* . Inhibition of C5aR1 also resulted in behavioral abnormalities in both sexes and MRI-detected brain microstructural alterations in adult male, suggesting that C5aR1 played a functional role in neurogenesis in mammals and provided mechanistic insight into complement-related brain disorders [327].

#### 2.8.3. The role of C5aR1 in diseases

##### The role of C5aR1 in non-CNS disease

The function of C5aR1 in various diseases was explored using cells or tissues from C5aR1 genetic knock-out mice or using a C5aR1 inhibitor or siRNA to inhibit C5aR1 expression [312, 328-331]. C5aR1 was implicated in a model of angiotensin II-induced hypertension. Genetic depletion of C5aR1 or pharmacologic inhibition of C5aR1 diminished hypertension-induced cardiac inflammation and remodeling [332]. In a mouse model of kidney ischemia, inhibiting C5aR1 via administering C5aR1 siRNA diminished neutrophil influx and cell necrosis in the renal tissue [333]. The protective effect of inhibiting C5aR1 during ischemic pathological conditions was demonstrated in a rat model of acute limb ischemia-reperfusion, rodent intestinal ischemia-reperfusion, and rat hepatic ischemia-reperfusion [334-338]. These studies demonstrated that pretreatment with a C5aR1 antagonist could diminish the production of inflammatory cytokines and prevent local and remote organ injury after ischemia [334-337]. The above studies suggest that C5aR1 plays a crucial role in mediating the inflammatory response under pathological conditions. The selected C5aR1 antagonist could be a worthwhile option to attenuate the inflammatory response and exert beneficial effects in multiple disorders, especially ischemia-reperfusion injury.

##### The role of C5aR1 in CNS disease

C5aR1 antagonist treatment could block neutrophil extravasation into the brain parenchyma after traumatic brain injury or intracerebral hemorrhage, contributing to reduced tissue damage and improved spatial memory after brain injury [338, 339]. The positive effect of C5aR1 inhibition on outcomes was found in an animal AD model. Genetic knock-out of C5aR1 or the specific C5aR1 antagonist reduced fibrillary plaque accumulation, inhibited microglial inflammatory polarization, and suppressed cognitive loss in mice [331, 340]. However, the molecular mechanism of C5aR1 and its downstream effector in the ischemic brain is unclear. Several studies have shown that C5aR1 expression is upregulated during ischemic brain injury. In a mouse model of permanent focal ischemia, C5aR1 mRNA expression was increased after 3 hours up to 21 days post-ischemia. C5aR1 was located on endothelial cells after 12 hours of ischemia and strongly expressed on reactive astrocytes and macrophage-like cells in the peri-infarct region of the ischemic cortex after 7 days of ischemia [173]. The elevation of C5aR1 expression in the ischemic brain within 24 hours of ischemia was similar in rodents. Increased C5aR1 was primarily observed in the meninges and outer cerebral cortex and on neurons in the granular layers after arterial occlusion [341]. In a mouse model of 1-hour MCAO, C5aR1 deficiency or C5aR1 antagonist treatment reduced brain infarct volume and improved neurological function. C5aR1 deficiency or C5aR1 antagonist treatment also protected cultured neurons from OGD-induced apoptosis [341]. In a rat model of hypoxic- ischemic encephalopathy, C5aR1 was predominantly expressed in microglia. Therapeutic hypothermia (HT) combined with the inhibition of the expression of C5aR1 could reduce brain infarct volume within 3 days of brain ischemia [342]. These studies suggested that C5aR1 activation resulted in deleterious consequences at the early phase of ischemic brain injury, raising an opportunity to use specific C5aR1 antagonists to modulate complement activation and attenuate acute ischemic brain injury in humans. Although the time course of C5aR1 expression was examined and its detrimental role at the early stage of ischemic stroke was demonstrated, the role of C5aR1 in the recovery process has not yet been explored. Future studies should allocate more effort to elucidate the role of C5aR1 at the later stage of cerebral ischemia, which provides new insights into potential strategies targeting C5aR1 during ischemic stroke.

### 2.9. C5aR2 in ischemic stroke

C5aR2, previously known as C5L2, is another receptor of C5a and C5a degradation product C5adesArg, the predominant form of circulating C5a [245, 343]. C5aR2 has a similar structure and cellular expression pattern of C5aR1 and is often coexpressed with C5aR1 [252, 344]. However, unlike C5aR1, C5aR2 is unable to couple to heterotrimeric G proteins and does not trigger the intracellular Gα signal [345]. As a consequence, C5aR2 functioned as a decoy receptor and limited the C5a availability [346]. However, increasing evidence has shown that C5aR2 is also a functional receptor and exerts both anti-inflammatory and pro-inflammatory effects in diverse disease models. C5aR2 deficiency led to enhanced inflammatory effects induced by C5a in rodent models of complex lung injury, allergic contact dermatitis, experimental allergic asthma, anti-neutrophil cytoplasmic antibodies (ANCA), and crescentic glomerulo-nephritis, suggesting an anti-inflammatory role for C5aR2 in these models [347-351]. In contrast, the absence of C5aR2 led to reduced inflammation in models of air pouch, acute lung injury, sepsis and renal ischemia-reperfusion injury in mice [256, 352-354] . Notably, C5aR2 was required for the production of pro-inflammatory mediators from human mast cells [355]. C5aR2 was also involved in the production of G-CSF in a mouse model of polymicrobial sepsis, which was characterized by acute inflammation [356]. Thus, the function of C5aR2 was probably mediating inflammation and immunity [250]. Although great progress has been achieved in the study of C5aR2 functions *in vitro* and in a variety of experimental animal models, little is known about its role in the CNS, including ischemic stroke. Therefore, the role of C5aR2, within and beyond the CNS, is a major area of complement research in the future [54].

## 3. Therapeutic approaches against complement in ischemic stroke

It is widely accepted that deregulated or excessive complement activation is involved in numerous diseases or pathological conditions, including ocular pathologies, kidney disease, hematological system disorder, cancer, neurodegenerative disorder, and ischemic stroke [94, 130, 357]. Thus, it spurred robust interest in the modulation of the complement system as a therapeutic option in drug discovery [43, 240, 358]. A large number of therapeutic strategies targeting various steps of the complement cascade were developed, including antibodies and fusion proteins, small interfering RNAs, aptamers, low-molecular-weight peptides, and non-peptide inhibitors [359-362]. Several agents that inhibit all or part of the complement system, such as C1 inhibitor (C1-INH), CVF, a C3aR antagonist, small or large molecule C5aR antagonists, and sCD59 could reduce ischemia/reperfusion brain injury [130, 239]. Next, we analyze the effect of different complement inhibitors and some possible mechanisms against ischemic brain injury.

**Table 1:** C1-INH treatment for ischemic stroke

Animals	Stroke models	Time of administration	Routes of injection	Time point of observations	Outcomes	Mechanisms	Refs
C57BL/6 mice	1 h MCAO	0.5 h before or 6 h after MCAO	Intravenous injection with IVIg or alone	72 h after MCAO	Reduced brain infarction size, neurological deficit and mortality	Reduced deposition of C3b and downregulated excessive TLR2 and p-JNK1 expression in the brain	[400]
C57BL/6 mice & CD rats	1 h MCAO (mice) & 1.5 h MCAO (rats)	1 h or 6 h after MCAO	Intravenous injection	24 h and 7 days after MCAO	Reduced infarct volumes and improved clinical scores	Reduced blood-brain barrier damage, edema formation, and inflammation	[366]
Sprague-Dawley rats	pMCAO or thromboembolic stroke	2 h or 4 h after MCAO	Intravenous injection with tPA or alone	24 h after MCAO	Reduced intracranial hemorrhage and neurological scores	Not studied	[428]
C57Bl/6 mice	0.5 h MCAO or pMCAO	0 h, 3 h, 6 h, or 18 h after MCAO	Intravenous injection	48 h and 7 days after MCAO	Reduced brain infarct volumes and CD45^+^ cell infiltration in the brain	Not studied	[367]
CD1 mice	2 h MCAO	Immediately after ischemia	Intravenous injection	48 h after MCAO	Attenuated general and focal neurological deficits	Reduced TNF-a, IL-18, ICAM-1 and P-selectin mRNA expression and microglia activation; Enhanced IL-6 and IL-10 mRNA expression	[369]
C57BL/6	0.5 h MCAO	15 min after ischemia	Intravenous injection	4 days after MCAO	Reduced general and focal neurological deficit scores and neuronal degeneration	Reduced CD45^+^ cell infiltration in the brain	[33]
CD1 mice	2 h MCAO	15 min after MCAO	Intravenous injection	48 h after MCAO	Reduced infarct volume and neuronal death	Did not affect astrocyte response	[364]
Wistar rats	1 h MCAO	Just before MCAO	Intravenous injection	48 h after MCAO	Reduced infarct volume and leukocyte infiltration in the brain	Not studied	[429]

### 3.1. Inhibition of complement initiation

#### 3.1.1. C1-INH

It is the interaction between C1q and serine proteases C1r and C1s that initiates the classical complement system pathway [43]. Under physiological conditions, the activity of the subcomponents of C1r and C1s is controlled by the plasma protease C1-INH, which has been registered in European markets since 1997 [363, 364]. C1-INH is the first clinical complement inhibitor and inhibits both classical and lectin pathways during the complement cascade [13, 43]. In a variety of experimental ischemic stroke studies, C1-INH treatment reduced brain infarct volume and attenuated neurological deficit during the acute and later stages of stroke [359, 365, 366]. Additional human recombinant (*rh* ) C1-INH was developed and had a wider effect in reducing ischemic brain injury than plasma-derived C1-INH [367]. The protective effect of C1-INH on stroke outcomes was mainly through inhibiting leukocyte infiltration and myeloperoxidase activity and diminishing neuronal apoptosis [33, 367-369]. The detailed information of C1-INH for ischemic stroke therapy is summarized in Table 1.

### 3.2. Inhibition of the amplification loop

#### 3.2.1. C3 inhibitor: CVF

C3 is in the central position of the complement cascade and is generated through three pathways of complement activation [370]. C3b, the active fragment of C3, plays a key role in forming C3 convertase and implies the complement response [94]. CVF is a structural homolog of C3b/C3c found in the venom of certain cobra species [371]. It is capable of binding factor B to form stable C3/C5 convertases that hydrolyze C3 and C5 in an uncontrolled manner, causing their depletion [372]. For almost four decades, CVF was used in animal models to explore the role of complement in a variety of disease states, including ischemia/reperfusion injury [373-375]. In experimental ischemic stroke, pretreatment with CVF could inhibit complement activation by removing circulating C3 and other complement components, exerting beneficial effects on the outcomes of ischemic brain injury [359]. CVF administration 24 hours before surgery resulted in reduced complement activity, combined with enhanced somatosensory-evoked potentials in a rat model of forebrain ischemia [376]. Pretreatment with CVF reduced ischemic brain injury in both adult and neonatal rats [64]. Furthermore, CVF pretreatment also reduced neuronal C3 deposition in the brain, despite no reduction in microglial C3 deposition and neuronal C9 deposition [63]. However, in a rabbit thromboembolic stroke model, pretreatment with CVF did not affect cerebral blood flow or brain infarct volume within 7 hours of embolization. It is noted that the injection of CVF immediately or 72 hours after cerebral ischemia showed no effect on attenuating brain edema and atrophy after 48 hours of brain injury [377]. These inconsistent results could be due to the distinct administration time of CVF and the difference in the pathophysiological process between adults and neonates (Table 2). Thus, it is possible that CVF served as a therapeutic agent when administered before ischemia. Studies are still warranted to clarify the therapeutic time window and mechanism of CVF in the pathogenesis of ischemic stroke to facilitate clinical transformation.

#### 3.2.2. sCR1

An alternative approach to broadly inhibit the amplification loop during complement activation is the recombinant form of endogenous complement C3 convertase inhibitor, sCR1. It is more effective to inhibit complement activation than other endogenous soluble C3/C5 convertase inhibitors, such as factor H and C4-binding protein (C4bp) [378]. sCR1 is the soluble extra membranous part of CR1, is shed from leukocytes, and retains full regulatory activity in plasma. It exerted an inhibitory function of spontaneous complement activation via decaying the C3 and C5 convertases and was elevated in plasma during the progression of certain diseases [379-381]. Numerous studies have shown that the application of sCR1 significantly alleviated complement-mediated tissue damage after ischemia/reperfusion injury [378, 382-386]. In a rat model of myocardial ischemia/reperfusion injury, sCR1 treatment reduced myocardial infarction and leukocyte infiltration within the infarct zone [378]. In a rat model of liver ischemia/reperfusion, intravenous injection of sCR1 24 hours after ischemia reduced complement activity and C3 deposition on endothelial cells and ameliorated the reperfusion injury [384]. In addition, sCR1 was considered a promising anti-inflammatory therapeutic agent to alleviate complement-mediated ischemic brain injury [387]. In a rat model of cerebral ischemia, intravenous administration of the SCR1-3 or the SCR15-18 functional domain of sCR1 1 hour before surgery reduced infarct volume, C3b deposition and neutrophil infiltration in the brain and attenuated motor deficits after 24 hours of ischemia [387, 388]. Furthermore, sCR1-sLex (sLex-glycosylated sCR1 modification) treatment showed better neuroprotective effects than unmodified sCR1 treatment in ischemic stroke [24]. However, in a baboon model of ischemic stroke, pretreatment with sCR1 or sCR1-sLex did not affect infarct volume or neurological score, although serum complement activity was significantly depressed [389, 390]. These studies suggested that the complement cascade in ischemic stroke was different among species. It is better to use the primate ischemic model to evaluate the therapeutic effect of sCR1 prior to the implementation of large-scale clinical trials.

In addition to the beneficial effects of sCR1 on models of ischemic diseases, sCR1 also displayed good therapeutic effects on animal models of reverse passive Arthur reaction, complement-mediated experimental glomerulonephritis, acute lung injury, adult respiratory distress syndrome (ARDS), demyelinating experimental allergic encephalitis, allogeneic lung transplantation and arthritis [391-397]. Clinical trials of sCR1 were performed in patients with acute lung injury and respiratory distress syndrome and showed well-tolerated and effectively inhibited complement activation [398]. It will take time to translate the clinical application of sCR1 for the treatment of ischemia stroke. More efforts must be paid in the future to understand the cellular and molecular mechanism of sCR1, which would accelerate the clinical translation of sCR1 for the treatment of cerebral ischemia.

**Table 2:** CVF administration for ischemic stroke.

Animals	Stroke models	Time of administration	Routes of injection	Time point of observations	Outcomes	Mechanisms	Refs
Adult rats & 7-day-old rats	1.5 h MCAO in adult rats; Unilateral carotid artery ligation followed by transient hypoxia in neonates	24 h before surgery	Intraperitoneal injection	48 h after surgery	Reduced brain infarct volume and brain atrophy	Not studied	[64]
7-day-old rats	Cerebral hypoxic-ischemic injury	24 h before surgery	Intraperitoneal injection	1 day and 5 days after cerebral ischemia	Reduced systemic C3 and neuronal C3 deposition in the brain; Reduced brain infarct size	Did not affect neuronal C9 deposition and microglial C3 deposition in the brain	[63]
7-day-old rats	Unilateral carotid artery ligation followed by transient hypoxia	24 h before surgery	Intraperitoneal injection	24 h after surgery	Did not reduce infarct volume	Not studied	[300]
Sprague-Dawley rats	Autologous blood induced intracerebral hemorrhage	36 h, 24 h, and 12 h before induction of intracerebral hemorrhage	Intraperitoneal injection	2 h, 24 h, or 72 h after intracerebral hemorrhage	Reduced brain water content and MPO activity at 72 hours after intracerebral hemorrhage	Not studied	[430]
21-day-old rats	Right common carotid artery ligation and hypoxia	24 h immediately before and 72 h after surgery	Intraperitoneal injection	24 h, 48 h after cerebral ischemia	Did not did not reduce the neuronal loss, brain edema or atrophy	Not studied	[377]
New Zealand white rabbits	Thromboembolic stroke	48 h before surgery	Intraperitoneal injection	7 h after surgery	Did not reduce brain infarct volume	Not studied	[431]
Sprague-Dawley rats	Reversible incomplete forebrain ischemia	24 h before surgery	Intraperitoneal injection	Within 4 h after cerebral ischemia	Had a tendency to increase focal cerebral blood flow and increased somatosensory evoked potentials in the cortex	Not studied	[376]

#### 3.2.3 IVIg (Table 3)

IVIg is a purified concentrated human immunoglobulin solution composed primarily of IgG extracted from the plasma of thousands of healthy donors [12, 399]. It was FDA approved and clinically used as a first-line therapeutic modality for diverse autoimmune and several inflammatory diseases [12]. Recently, experimental studies have shown that IVIg has a beneficial therapeutic potential in acute brain injury, including trauma, subarachnoid hemorrhage, and ischemic stroke [12]. As a scavenger of activated complement fragments, IVIg could inhibit complement activation and attenuate brain damage in a rodent stroke model [269]. In a mouse model of 1-hour MCAO, IVIg injection via the femoral vein 0.5 hour before or 1 hour after the surgery reduced brain infarct area, mortality, and neurological deficit score at 72 hours after ischemia. In addition, IVIg also inhibited C3b deposition and TLR2 expression in the brain [400]. IVIg reduced neuronal apoptosis induced by OGD via inhibiting the upregulation of C3 and activation of caspase-3 [269]. These studies suggested that IVIg was neuroprotective by modulating complement activation during ischemic conditions. The anti-inflammatory effect of IVIg was reducing TLR expression, suppressing high mobility group box 1 (HMGB1)-mediated TLR activation, and reducing the levels of the inflammatory components NLRP1 and NLRP3 [401, 402]. *In vitro* studies showed that IVIg protected neurons and endothelial dysfunction by inhibiting p38MAPK, c-Jun NH2-terminal kinase, p65 phosphorylation, and dampening the decline in the anti-apoptotic proteins Bcl-2 and Bcl-XL induced by OGD [401, 403, 404]. Apart from the described pathways, multiple conceivable mechanisms were involved in how IVIg facilitated protection in ischemic stroke [12]. For example, IVIg could induce a regulatory phenotype in macrophages in an Fc-fragment manner and target the inhibitory receptor FcγRIIB, subsequently suppressing T cell-mediated immune responses during ischemic stroke. Therefore, IVIg is a promising therapeutic for ischemic stroke. Because IVIg treatment was mostly used in the experimental studies, it should be studied in clinical stroke patients in the future.

**Table 3:** IVIg treatment for ischemic stroke.

Animals	Stroke models	Time of administration	Routes of injection	Time point of observations	Outcomes	Mechanisms	Refs
C57BL/6 mice	1 h MCAO	0.5 h before or 1 h, 3 h and 6 h after surgery	Intravenous injection of IVIg (1 g/kg)	72 h after MCAO	Reduced brain infarct area, mortality, and neurological deficit score	Inhibited C3b deposition and TLR2 expression in the brain	[400]
C57BL/6J mice	1 h MCAO	3 h after surgery	Intravenous injection of IVIg (1 g/kg)	6 h, 24 h after MCAO	Increased low-density lipoprotein receptor-related protein 1 (LRP1) tyrosine phosphorylation in the brain	Inhibited pro-death signaling pathways such as NF-κB, MAPKs, and caspase-3 in cultured neurons under OGD condition	[432]
Sprague-Dawley rats	2 h MCAO	Just after surgery	Intravenous injection of IVIg (400 mg/kg)	72 h after MCAO	Reduced neurological deficit score	Not studied	[433]
C57BL/6 mice	1 h MCAO	3 h after surgery	Intravenous injection of IVIg (1 g/kg)	6 h, 24 h after MCAO	Reduced TL2, TLR4 and TLR8 expression in the brain; Reduced NF-κB and MAPK activities in the brain	Inhibited HMGB1 induced activation of NF-κB-p-p65, p-JNK, p38 MAPK and p-c-Jun, and increased Bcl-2 expression in cultured neurons under oxygen and glucose deprivation	[403]
C57BL/6 mice	1 h MCAO	3 h after surgery	Intravenous injection of IVIg (2 g/kg)	24 h after MCAO	Reduced CD45^+^ leukocyte infiltration in the brain	Not studied	[401]
C57BL/6J mice	1 h MCAO	3 h after surgery	Intravenous injection of IVIg (1 g/kg)	6 h, 24 h, 72 h after MCAO	Reduced inflammasome components NLRP1 and NLRP3, and IL-1β and IL-18 expression in the brain	Not studied	[402]
C57BL/6 mice	1 h MCAO	0.5 h before or 3 h after surgery	Intravenous injection of IVIg (2 g/kg)	24 h after MCAO	Reduced brain infarct volume and neurological deficit, increased neuronal survival	Inhibited phosphorylation of the cell death-associated kinases p38 MAPK, JNK and p65 in cultured neurons under OGD condition	[404]
Wistar rats	1.5 h MCAO	0.5 h before surgery	Intravenous injection of IVIg (1 g/kg)	24 h after MCAO	Reduced brain infarct volume and neurological deficit score	Not studied	[434]
C57BL/6 mice	1 h MCAO	0.5 h before or 3 h after surgery	Intravenous injection of IVIg (2 g/kg)	24 h after MCAO	Reduced brain infarct volume and neurological deficit	Inhibited C3 elevation in the brain; Reduced endothelial cell adhesion, lymphocyte infiltration, and microglial activation	[269]

**Table 4:** sCR1 treatment for ischemic stroke.

Animals	Stroke models	Time of administration	Routes of injection	Time point of observations	Outcomes	Mechanisms	Refs
Sprague-Dawley rats	1 h MCAO	1 h before surgery	Intravenous injection of sCR1	1 h, 24 h after surgery	Reduced brain infarct volume and neurological motor deficits	Reduced neutrophil accumulation and inflammation; Reduced C4b deposition in the cortex	[387]
Sprague-Dawley rats	2 h MCAO	1 h before surgery	Intravenous injection of sCR1	2 h, 24 h after surgery	Reduced brain infarct size and neurological deficit scores	Inhibited neutrophil infiltration and C3b deposition in the brain	[388]
Adult male baboons	1.25 h MCAO	45 min before surgery	Intravenous injection of sCR1-sLex	2 h, 6 h, 12 h, 72 h and 10 days of post-ischemia	Increased brain infarct volume; Did not improve neurological functions	Not studied	[390]
Adult male baboons	1.25 h MCAO	45 min before surgery	Intravenous injection of sCR1	72 h and 10 days of post-surgery	Did not affect brain infarct volume and neurological scores	Not studied	[389]
7-day-old rats	Unilateral carotid artery ligation followed by transient hypoxia	24 h before surgery	Intraperitoneal injection of sCR1 or sCR1-sLex	24 h after surgery	Did not reduce infarct volume	Not studied	[300]
Mice	0.75 h MCAO	Immediately before MCAO	Administration of sCR1 or sCR1-sLex	24 h after stroke	Reduced brain infarct volume and neurological deficit score	Inhibited neutrophil and platelet accumulation in the brain	[24]

### 3.3. Inhibition of effector function (Table 4)

#### 3.3.1. The C3aR antagonist

C3aR is the receptor of C3a, which is generated from C3 after being cleaved by C3 convertase. As one of the terminal mediators of the complement system, genetic depletion or a specific antagonist of C3aR was used to define the role of C3aR and modulate its activity during different kinds of diseases, including intestinal ischemia/reperfusion injury, myocardial ischemia and reperfusion injury, hypertension, brain death induced by lung injury, kidney disease, type 2 diabetic nephropathy, arthritis, AD, intracerebral hemorrhage, and ischemic stroke [34, 67, 172, 174, 339, 405-413]. Studies have demonstrated that C3aR antagonists emolliate ischemic brain injury, mainly through reducing neutrophil infiltration and the inflammatory response at the acute stage of ischemic stroke [34, 174]. However, it is still under debate whether C3aR antagonists are beneficial or detrimental for the promotion of neurogenesis after ischemic stroke. In a transient ischemic stroke study, a low dose of C3aR antagonist (1 mg/kg) within 3 days after ischemia increased DCX^+^ neuroblast proliferation in the subventricular zone at 7 days post-ischemia, while a high dose of C3aR antagonist (40 mg/kg) suppressed neural progenitor cell proliferation [414]. The opposite results were observed in a mouse permanent ischemia model. C3aR antagonist injection twice daily for 10 days after surgery inhibited neuroblast proliferation in the subgranular zone (SGZ) of the hippocampus and in the dentate gyrus granule cell layer (GCL) at 7 to 21 days of post-ischemia [67]. The discrepancy of these studies could be the result of the distinct pathological response of the whether stroke was blood-perfused and the different doses and regimens of the C3aR antagonist injection [415]. To better optimize the therapeutic strategies modulating C3aR activity during the pathological process of cerebral ischemia, different doses, injection routes, and injection time-points should be explored in the future in different models of ischemic stroke.

#### 3.3.2. Anti-C5 monoclonal antibodies and the C5aR1 antagonist

C5a is the active fragment of C5 and exerts its pathophysiological functions via binding with its receptor C5aR. As another key downstream effector of the complement system, the interaction between C5a and C5aR1 was widely recognized as a pro-inflammatory signaling in multiple diseases [335]. Both anti-C5 monoclonal antibodies and C5aR1 antagonists were developed and used as therapeutic agents to attenuate tissue injury in various rodent disease models, including kidney ischemia/reperfusion injury, intestine ischemia/reperfusion injury, sepsis, spinal cord injury, CNS lupus, hypertension, AD, intracerebral hemorrhage, and ischemic stroke [270, 339, 340, 416-423]. In a rat model of ischemic stroke, administration of the anti-C5 monoclonal antibody, specifically blocking the generation of C5a and C5b-9, reduced brain infarct volume and edema and improved neurological function at 24 hours after ischemia [270, 424]. Similarly, pre- or posttreatment with the C5aR1 antagonist AcF, also known as PMX53, reduced cerebral infarct volume at 24 hours of ischemic stroke in mice [425]. These studies suggested that the modulation of C5a/C5aR signaling was a beneficial potential at the early stage of ischemic stroke. However, the effect of anti-C5 monoclonal antibodies and C5aR1 antagonists in ischemic stroke is limited. Whether they still exert protective roles at the later stage of ischemic stroke and what mechanism they work through are both poorly investigated. Therefore, developing better strategies for modulating C5a/C5aR activities in ischemic stroke before the application of potential candidates to clinical practice is greatly needed.

## 4. Challenges and prospective

### 4.1. Evolving challenges

Although the value of inhibiting complement in a therapeutic context of ischemic stroke in experimental studies has long been recognized, bringing complement-targeted drugs into clinical use has proved challenging. While a broad variety of candidate drugs that target various stages of the complement cascade are currently being evaluated in clinical trials, they cannot be applied to all complement-driven diseases [43]. The available therapeutics modulating the complement system for stroke patients are still limited [426]. Therefore, challenges remain and must be overcome before the translation of complement-targeted therapeutics for the treatment of clinical ischemic stroke is achieved. It is important to avoid infectious complications and preserve the physiological and regenerative functions of different components during the pathogenesis of ischemic stroke when using systemically active complement-blocking agents. First, as several components, such as C1q, C3, and C3aR, are involved in synaptic plasticity and neurogenesis under both normal and stroke conditions, it is imperative to choose more specific and focused complement modulation, which may cause fewer undesired complications [359, 427]. The timing, dose, and duration of treatment (transient or chronic) also require careful consideration since they are all likely factors affecting the efficiency of therapeutic strategies. Second, the use of traditional small molecules or biologic approaches to modulate the complement system is imperative. The majority of emerging complement therapeutics commercially available are antibodies or proteins, which are too large to cross the intact BBB [359]. Although they may prove effective in acute brain injury such as ischemic stroke where the BBB is disrupted, their pharmacologic effects are compromised and decline at the chronic phase of ischemic stroke or during chronic cerebral ischemia. In addition, choosing therapeutic agents whose circulating levels are very high and whose turnover rates are relatively rapid is required to sustain benefits for patients with chronic cerebral ischemia [427]. A third challenge is linking the discoveries of the changes in complement components and effector mechanisms causing tissue injury in both experimental and clinical ischemic stroke. The determination of the consistency between basic research and clinical studies is the foundation of applying therapeutics targeting complement systems into clinical use. Last but not least, it is important to choose the right therapy that is the most appropriate for each individual patient if complement-targeted therapeutics are to be successful in clinical trials. As a biomarker, complement activation in plasma could inform the appropriate selection of treatment for individual patients and help identify optimal therapeutics.

### 4.2. Future Perspective

It is undeniable that the complement system is implicated in the pathophysiology of ischemic stroke. However, our understanding and insight into the cascade mechanisms and their complex interaction with neuronal cells in the brain after ischemic stroke is still in its nascent phase. Currently, the predominant focus of studies on complement and ischemic stroke is on the role of complement in eliciting the inflammatory cascade and contributing to tissue injury. The majority of studies mainly investigated acute-phase outcomes, where complement depletion or inhibition prevented the activation of pro-inflammatory mechanisms and the deficit of neurological functions. By contrast, few studies have explored the role of the complement system in subacute and chronic phases after ischemic stroke, when complement components could be important for regeneration and remodeling. An investigation of the temporal and spatial changes, as well as the role of different complement components at subacute and chronic stages after stroke, is useful for guiding therapeutic strategies targeting the complement system and reducing side effects. Therefore, future studies should design experimental models and evaluate outcomes for longer endpoints after stroke. Although the essential role of complement in synaptic pruning, loss, and remodeling during CNS development and neurodegenerative conditions has been demonstrated, it is unclear whether complement is also necessary in synaptic loss or remodeling in the early and delayed pathophysiological processes of ischemic stroke [206, 207, 213]. In addition, whether serum-derived complement and resident cells in brain-derived complement have the same or distinct impacts on ischemic stroke outcomes remains ambiguous. Addressing these unanswered questions could reveal a more diverse role for complement in ischemic brain injury and rescue.

In summary, numerous preclinical studies have demonstrated that the complement system acts as an important player involved in tissue injury and recovery. Consequently, a wealth of complement-targeted therapeutics has been developed to modulate complement activation and attenuate tissue injury. Much effort is still needed to elucidate the precise role of different complement components in the pathophysiological process of ischemic stroke. In the next decades, there will be dramatic improvements in developing novel therapeutic candidates with potentially good safety profiles for the treatment of ischemic stroke.
